# Therapeutic options for premature ovarian insufficiency: an updated review

**DOI:** 10.1186/s12958-022-00892-8

**Published:** 2022-02-04

**Authors:** Qiao-yi Huang, Shao-rong Chen, Jia-ming Chen, Qi-yang Shi, Shu Lin

**Affiliations:** 1 Department of Gynaecology and Obstetrics, The Second Affiliated Hospital of Fujian Medical University, No.34 North Zhongshan Road, Quanzhou, 362000 Fujian Province China; 2 Centre of Neurological and Metabolic Research, The Second Affiliated Hospital of Fujian Medical University, No.34 North Zhongshan Road, Quanzhou, 362000 Fujian Province China; 3 Diabetes and Metabolism Division, Garvan Institute of Medical Research, 384 Victoria Street, Darlinghurst, Sydney, NSW 2010 Australia

**Keywords:** Premature ovarian insufficiency, Therapeutic options, Stem cell therapy, In vitro activation, Mitochondrial activation technique, Platelet rich plasma

## Abstract

Primary ovarian insufficiency (POI) is a rare gynecological condition. This disease causes menstrual disturbances, infertility, and various health problems. Historically, hormone replacement therapy is the first-line treatment for this disorder. Women diagnosed with POI are left with limited therapeutic options. In order to remedy this situation, a new generation of therapeutic approaches, such as in vitro activation, mitochondrial activation technique, stem cell and exosomes therapy, biomaterials strategies, and platelet-rich plasma intra-ovarian infusion, is being developed. However, these emerging therapies are yet in the experimental stage and require precise design components to accelerate their conversion into clinical treatments. Thus, each medical practitioner bears responsibility for selecting suitable therapies for individual patients. In this article, we provide a timely analysis of the therapeutic strategies that are available for POI patients and discuss the prospects of POI therapy.

## Background

Primary ovarian insufficiency (POI), also referred to as premature ovarian failure (POF), pertains to the loss of ovarian function under the age of 40 years [1]. It is characterized by a decrease in ovarian follicles, and a dirth of hormone secretion. Although the prevalence of POI was previously reported as varying between 0.9–1.2% [2], a national register study conducted in Sweden has indicated that the total prevalence of POI approximates 1.9% [3]. A recent meta-analysis conducted by Golezar et al., estimated that 3.7% of women worldwide are affected [4]. POI patients experience long term complications, such as osteoporosis, fractures, cardiovascular diseases and depression [5]. Furthermore, this disease shows great potential for destroying the hopes and dreams of parenthood.

Diagnostic criteria for POI include oligo/amenorrhea for at least 4 months and two elevated follicle-stimulating hormone (FSH) levels (> 25 IU/L) more than 4 weeks apart [6]. A delay in diagnosing POI may cause irreversible impairment to the fertility of patients. Anti-Mullerian hormone (AMH) and antral follicle count (AFC) are other sensitive indicators are used to assess ovarian reserves.

POI is a heterogeneous disorder caused by genetic factors, autoimmune diseases, mitochondrial abnormalities, iatrogenic factors (including chemotherapy, radiotherapy, and surgical procedures), and environmental factors [7]. Additionally, a significant proportion of POI patients are idiopathic with undetermined etiology [8].

**Fig. 1:**
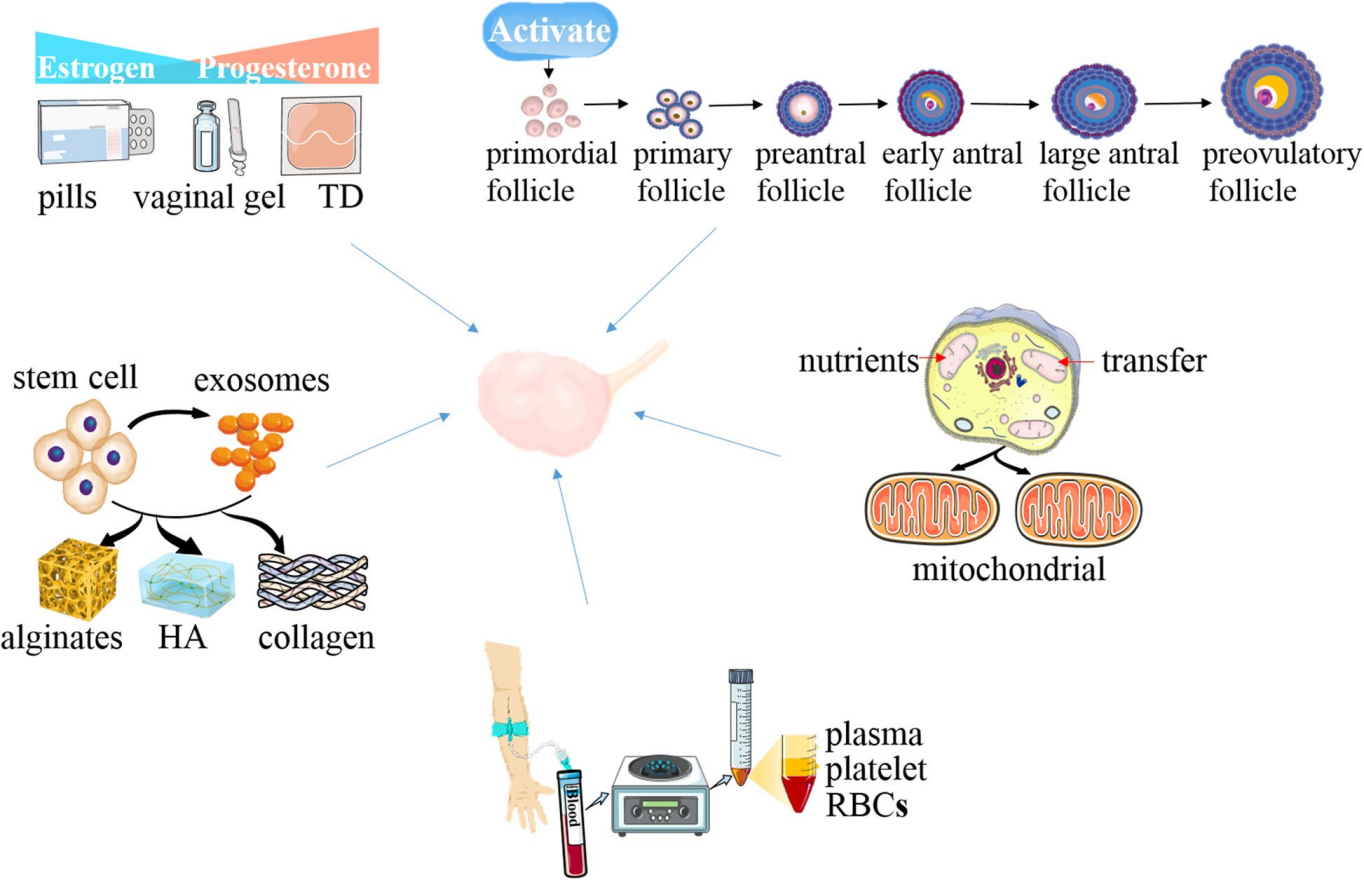
Several treatments of premature ovarian failure. For POI patients, HRT is the first-line treatment. In vitro activation, mitochondrial activation technique, stem cell and exosomes therapy, biomaterials strategies, and platelet-rich plasma intra-ovarian infusion are a new generation of treatments for POI management

POI may be treated in one of several ways (Fig. 1). Hormone replacement therapy (HRT) should be regarded as a physiological replacement of estrogens (+ progestin), but it fails to restore ovarian function. Currently used novel strategies mainly include in vitro activation (IVA), mitochondrial activation, stem cell and exosomes therapy, biomaterials strategies and intra-ovarian infusion of platelet-rich plasma (PRP). However, these new therapies, which are expected to be breakthrough therapies for POI, are still in their experimental stages, and their efficacy and safety must be proven prior to acceptance as true clinical options. This review summarizes current and future therapeutic strategies for POI.

## Innovative therapeutic options for POI

### In vitro activation

A previous study has indicated that approximately 75% of POI patients may carry residual dormant primordial follicles (PFs) in the ovaries [5]. The stored PFs of such patients may be effectively used to treat their infertility. Furthermore, IVA is a novel technique that stimulates the growth of PFs and induces these to develop into competent oocytes. In this manner, POI patients are able to produce offspring using their own genes.

#### Molecular regulation of IVA

**Fig. 2:**
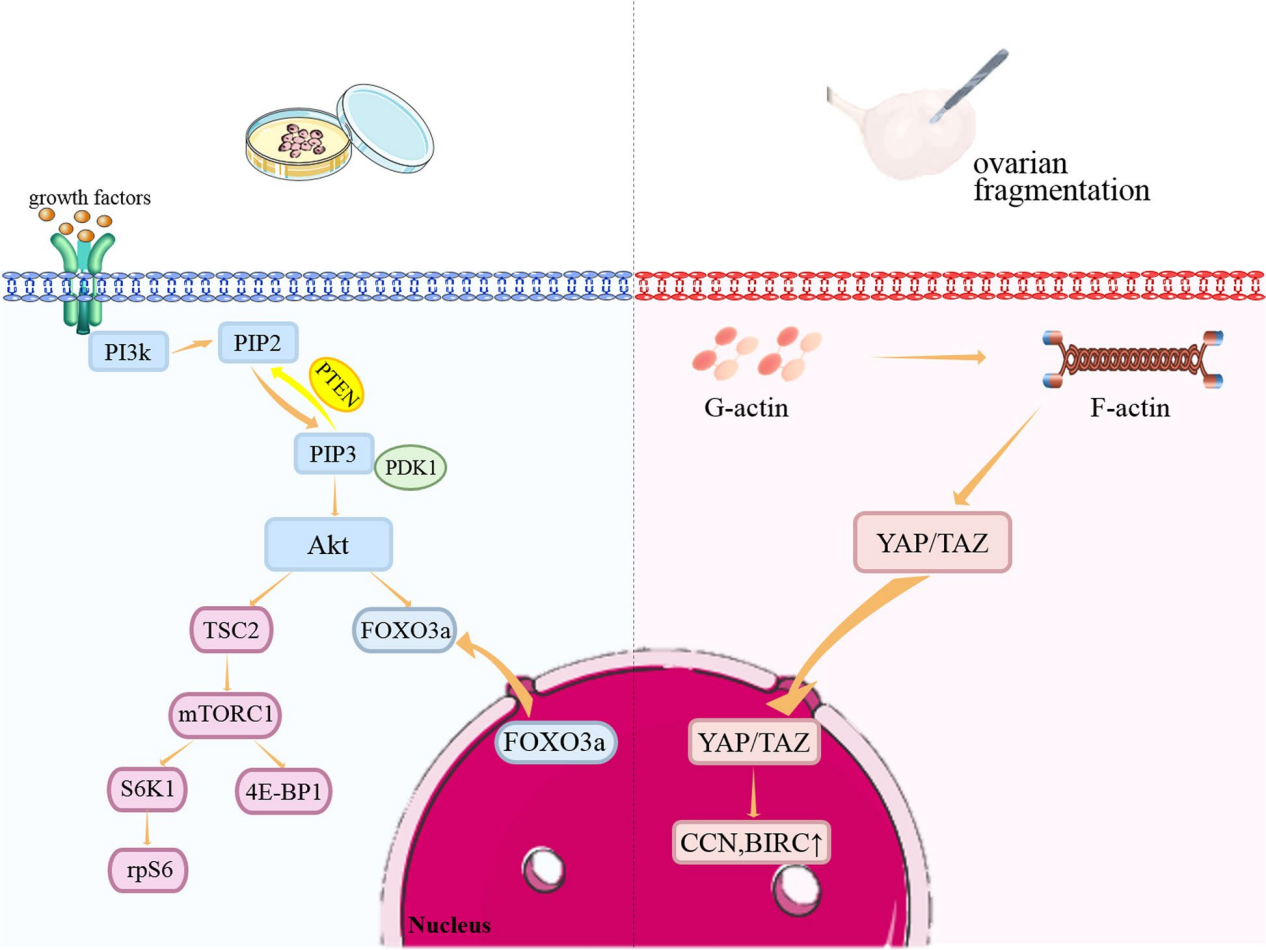
The PTEN/ PI3K / Akt / FOXO3 and Hippo signaling pathways regulates primordial follicles activation

IVA mainly involves phosphatase and tensin homolog (PTEN)/phosphatidylinositol-3-kinase (PI3K)/protein kinase B (Akt)/forkhead box O3 (FOXO3) signaling pathway, and the Hippo signaling pathways (Fig. 2). The PTEN/PI3K/Akt/FOXO3 pathway plays a central role in PFs activation. The cognate tyrosine kinase receptor initiates Akt activation signaling by enhancing PI3K activity, which converts secondary messenger, phosphatidylinositol-4,5-bisphosphate (PIP2), into phosphatidylinositol-3,4,5-triphosphate (PIP3). Subsequently, PIP3 activates phosphatidylinositol-dependent kinase 1 (PDK1), resulting in Akt activation [9], a key kinase in this pathway. Once Akt is activated, FOXO3a is hyperphosphorylated and undergoes nuclear exportation, eventually triggering PFs activation. Furthermore, Akt phosphorylates tuberous sclerosis 2 (TSC2), which inactivates TSC1/TSC2 complex and induces mammalian target of rapamycin complex (mTORC1). Subsequently, mTORC1 and its substrates promote PFs survival [10]. However, these events are reversed by the effects of PTEN, which negatively regulates this pathway by transforming PIP3 back to PIP2 [11].

The Hippo signaling pathway acts as a conservative regulator of organ size [12]. It consists of different negative growth regulators that inactivate Yes-associated protein (YAP)/transcriptional co-activator PDZ-binding motif (TAZ) signaling through phosphorylation. YAP/TAZ signaling plays a significant role in promoting the expression of intercellular signal proteins. In the ovary, mechanical signals, as ovarian fragmentation, disrupt the Hippo pathway by increasing the polymerization of G-actin into F-actin, resulting in the nuclear translocation of YAP [13]. The transcriptional interaction between nuclear YAP and TAZ increases the expression of CCN growth factor and baculoviral IAP repeat-containing (BIRC), leading to PF growth [14].

PFs activation involves multiple factors. Bone morphogenetic proteins (BMPs) and growth and differentiation factor 9 (GDF-9) are known to participate in early folliculogenesis. A study has indicated that BMP4 promotes the transformation of primordial follicles to primary follicles in a mouse ovary culture [15]. GDF-9 and BMP15 reportedly protect granulose cells (GCs) from apoptosis and improve the survival rate of follicles [16]. Moreover, activins can promote oocyte survival and PFs formation via the Smad2/Smad3 pathway [17]. By contrast, AMH is considered as an inhibitor of PFs activation. In AMH-cultured rat ovaries, PFs remained static despite the presence of other stimuli [18].

#### Preclinical studies of IVA

IVA is based on a large number of animal experiments. For example, Adhikari et al. [19] treated mouse ovaries with PTEN inhibitor bpV for 24 h and then transplanted them into the kidney capsules of the matched ovary to generate mature oocytes. The study also confirmed that transient treatment with PTEN inhibitors did not cause tumor formation or other chronic illnesses in recipient mice. Additionally, the number of antral follicles was increased in ovaries co-treated with MHY1485 (an mTOR activator) and Akt stimulators [10]. Recently, Zhang et al. revealed that topical injection of epidermal growth factor and bio-gel into ovaries could activate PFs by elevating CDC42-PI3K signaling and the PI3K/Akt pathway in murine as well as human ovarian tissues [20].

These studies indicated that PTEN/PI3Kb signaling regulation can be applied to reproductive practice. However, the impact of this technology on the survival and function of follicles has raised concerns. PTEN inhibition promotes PFs activation, and is accompanied by DNA damage and impaired DNA repair competence [21]. This result may be explained away as being due to the high metabolic activity and proliferation rate of GCs following Akt activation [22]. Another explanation pertains to impaired contact between somatic cells and oocytes [23].

Although fragmentation of ovaries disrupts the Hippo pathway, existing animal studies have indicated that differences may exist in the degree of ovarian fragmentation [24]. Furthermore, the connection between actin polymerization and the Hippo signaling pathway offers another potential target for IVA. Studies have reported that incubating ovaries with drugs that promote actin polymerization, such as jasplakinolide or sphingosine-1-phosphate, induces actin polymerization accompanied by an increase in nuclear YAP, leading to follicle growth [25].

In recent years, the role played by mechanical stress in the interaction between ovarian cells and the microenvironment has attracted much attention. Although the collagen-rich ovarian cortex provides a rigid physical environment that supports the follicle structure, ovarian extracellular matrix (ECM) rigidity limits the development and maturation of the follicle [26]. Furthermore, ECM digestion may induce the nuclear export of FOXO3a and oocyte growth [27]. Deposition and remodeling of mechanical matrix components, such as collagen, elastin, elastin microfibril interface localization protein-1, and fibrin-1, are associated with early follicle activation [28]. The above findings indicate therapeutic targets at the ECM level.

#### Translation of IVA in the practice of human reproduction

IVA has been successfully applied in clinical practice. Conventional IVA in POI patients was the combination of PTEN inhibitors and PI3K activators, followed by ovarian fragmentation and autografting cortical strips via laparoscopic surgery. This procedure resulted in two pregnancies and one healthy delivery [14]. In 2016, Zhai et al. reported a successful delivery after simplifying IVA by fresh tissue auto-transplantation [29].

Drug-free IVA was developed more recently. It focuses only on disrupting the Hippo pathway and avoids chemical activation of ovaries. A growing number of studies have reported that drug-free IVA had led to successful pregnancies [30, 31]. However, these results should be interpreted with caution because most of these studies, involved a limited number of patients and lacked controlled trials.

IVA provides a beneficial option for cancer patients because this technology increases the effectiveness of activating PFs via ovarian tissue freezing which maximizes the number of available oocytes [32]. After the primary disease is alleviated, the patients can transplant activated ovarian tissue based on their individual reproductive wishes. Notably, tumor cells may remain in the transplants of tumor patients, and thus the application of follicular separation technology and in vitro culture systems are utilized to minimize the risk of tumor enhancement. Finally, the necessity for IVA in fertility preservation has been questioned due to the spontaneous activation of PFs occurring in conventional fertility preservation [33]. In addition, another study has reported that excessive activation of follicles cause follicle loss and shortens the lifespan of graft [34].

### Mitochondrial activation technique

Mitochondria are the power sources of cells and the only organelles that contain a unique genome, termed mitochondrial DNA (mtDNA). Reportedly, mtDNA is a double-stranded, circular DNA with a length of approximately 16.5 kb, containing 37 genes encoding 13 protein, 2rRNAs, and 22 tRNAs [35]. The main function of mitochondria is to produce adenosine triphosphate (ATP) by oxidative phosphorylation [35]. In addition, mitochondria regulate other pathways, including calcium signaling, intracellular redox potential and apoptosis [36]. Mitochondria are also the primary source of intracellular reactive oxygen species (ROS). More importantly, the mitochondrial are tightly linked to oocytes quality and embryonic development.

#### Mitochondria and ovarian aging

Ovarian aging caused by mitochondrial dysfunction involves mtDNA dysfunction, enhanced oxidative damage, altered membrane potential and inefficient biogenesis or mitochondria clearance [37]. Of these, mtDNA dysfunction includes decreased mtDNA content, strand breaks, point mutations, and oxidative damage. Patients with POI reportedly exhibited significantly decreased mtDNA content compared to healthy fertile women [38]. Furthermore, mtDNA is prone to mutations due to the absence of histone protection and DNA repair enzymes [39]. Studies have shown that a single-point alteration in mtDNA profoundly influences mitochondrial proteostasis and reactive ROS generation, leading to telomere shortening [40]. Moreover, the introduction of mutated mtDNA polymerase gamma (POLG) into a mouse caused premature senescence [41].

ROS levels in POI populations are significantly higher [42]. Excessive accumulation of ROS drives mtDNA mutations and energy deficiency, consequently inducing aging [43]. In turn, mtDNA mutations further exacerbate the production of ROS. This vicious circle of self-amplification and destruction may lead to cell apoptosis. Additionally, overproduction of ROS may overwhelm cellular antioxidant defenses, leading to oxidative stress (OS) and premature aging [44].

Disturbances of mitochondrial dynamics, such as mitochondrial fusion, changes in mitochondrial metabolism, and imbalances in calcium homeostasis, also affect oocyte aging [43]. Mitofusin 2 (MFN2) is a key protein involved in mitochondrial fusion, causing oocytes lacking MFN2 that contributed to female infertility [45]. Mitochondrial fission factor dynamin-related protein 1(Drp1) is the key to maintaining oocyte quality. A previous study reported that Drp1 knockout may lead to follicular dysplasia and ovulation disorders [46]. Furthermore, the lack of mitochondrial proteases may lead to mitochondrial-related diseases and aggravate oocyte aging [47].

#### Mitochondrial nutrient therapy

In recent years, researchers have focused on using pharmacological methods to restore the vitality of mitochondria. Available mitochondrial nutrients include Coenzyme Q10 (CoQ10), resveratrol, melatonin, and rapamycin [48]. CoQ10 is a component of the mitochondrial electron transport chain and a cellular antioxidant, which reportedly delays the depletion of ovarian reserve [49]. Importantly, the only human trial conducted so far, has shown that supplementation with CoQ10 may reduce the rate of aneuploidy in oocytes after meiosis. However, the result of this study was not statistically significant due to the study being prematurely terminated due to safety considerations [50].

Resveratrol, an anti-aging compound, has been found to slow down ovarian aging and promote in vitro maturation of oocytes [48, 51]. However, resveratrol has not been recommended for routine clinical treatment due to an anti-deciduogenic effect that may reduce clinical pregnancy rate [52]. In addition, melatonin is considered to be an antioxidant against mitochondria. A published study showed that melatonin could delay ovarian aging in mice by increasing antioxidant capacity, maintaining telomerase activity and activating sirtuin1 [53].

#### Mitochondrial transfer therapy

Various mitochondrial transfer therapies have been tested for infertility management. A previous study has reported that allogeneic ooplasmic transfer in human oocytes led to successful pregnancy and live birth [54]. However, this technique was suspended due to the risk of heteroplasmy, and potential transmission of mitochondrial diseases, as well as Turner syndrome and autism cases being reported following transplantation [55]. Subsequently, new nuclear transplantation techniques, including spindle transplantation, germinal vesicle (GV) transplantation, and pronuclear transplantation (PNT), have been proposed.

Spindle transfer refers to the extraction of the spindle and its transplantation into an enucleated donor oocyte. In 2016, a study reported the first successful delivery by a woman with Leigh’s Syndrome (a rare mitochondrial disease) using this technique [56]. GV transfer, which improves meiotic resumption and oocyte maturation has been used against aneuploidy in infertile women [57]. However, it is noteworthy that mitochondria near the GV may be carried into reconstructed oocytes, leading to mitochondria heterogeneity, which adversely affecting offspring. Therefore, complete removal of mitochondria in patients is essential, although this remains a challenge in GV transfer. PNT involves the transfer of pronuclei from one zygote with abnormal mtDNA to another with healthy mtDNA. However, although PNT has led to triplet pregnancies [58], this technique is limited by ethical concerns due to the potential loss of zygotes.

Concerns regarding heteroplasmy resulted in the proposal of autonomous germline mitochondrial energy transfer (AUGMENT). The goal of AUGMENT is to isolate and obtain the mitochondria from oogonial stem cells and transfer them into the oocyte during intracytoplasmic sperm injection [59]. Oktay et al. revealed that women treated with AUGMENT showed high fertilization rates and embryonic scores. However, the authors failed to demonstrate a benefit for women > 40 years [60]. Furthermore, a well-designed random clinical trial questioned the effectiveness of AUGMENT, because women treated with AUGMENT showed lower mtDNA content and live birth rates than those in the control group [61]. Therefore, it may be concluded that current research on this procedure is preliminary at best, indicating that it is too early to apply AUGMENT to clinical practice.

### Stem cell therapy

Stem cell therapy is expected to restore ovarian function and fertility for POI patients. Stem cells are early, undifferentiated cells with the ability to self-renew, unlimited proliferation, and multi-differentiation. They are classified as embryonic stem cells (ESCs), adult stem cells (ASCs), and induced pluripotent stem cells (iPSCs) according to their origin [62]. Mesenchymal stem cells (MSCs) are a subset of ASCs isolated from multiple tissues, including bone marrow, adipose tissue, menstrual blood, umbilical cord, amniotic fluid, and placenta [63].

#### Stem cell therapy mechanisms in POI

**Fig. 3:**
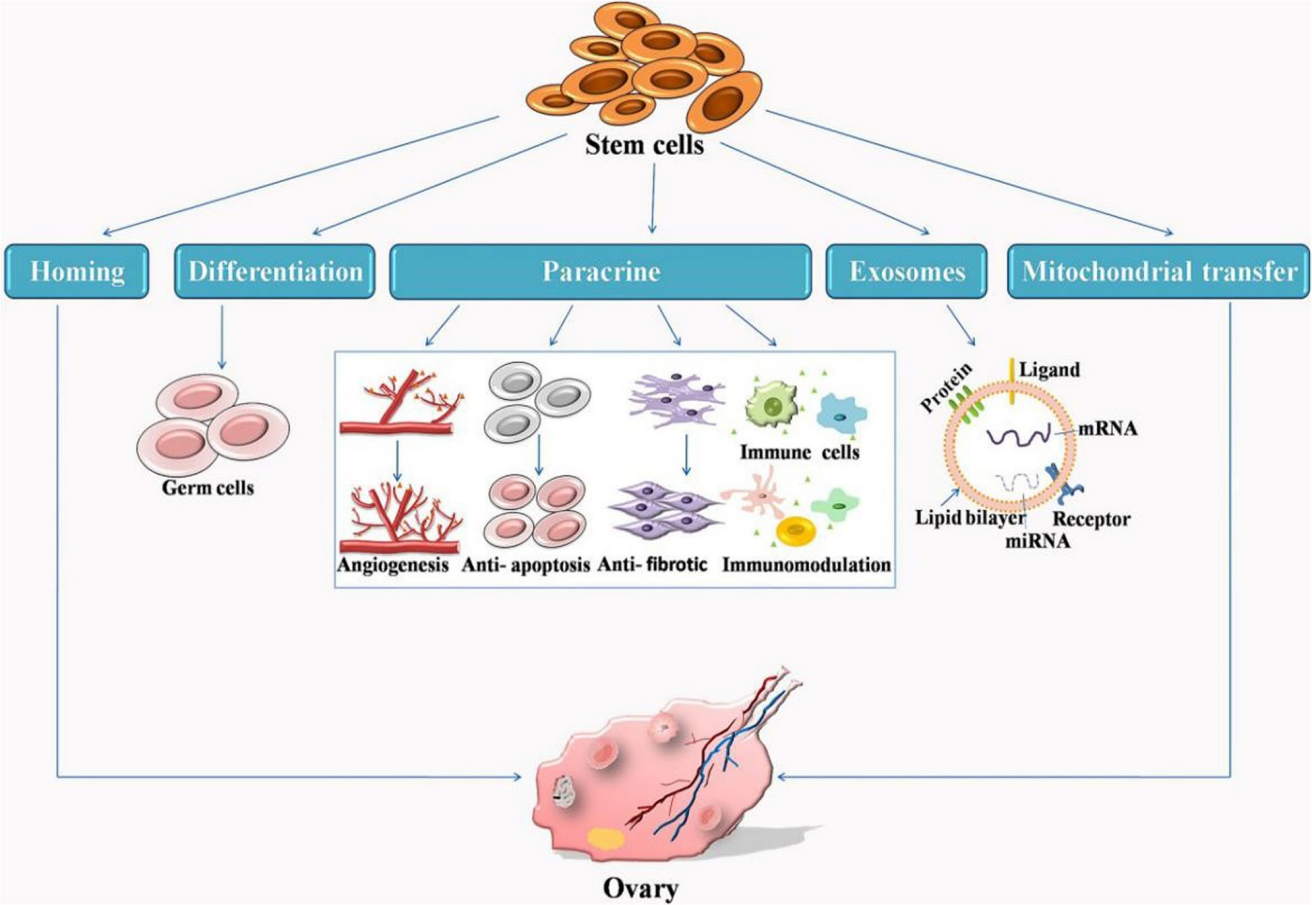
Stem cell therapy mechanisms in primary ovarian insufficiency. During ovarian insufficiency, stem cells play multiple roles through homing, differentiation and paracrine stimulation. Paracrine signaling is the key for the improvement of damaged ovaries through anti-apoptosis, anti-fibrotic, angiogenesis, anti-inflammation, and immune regulation. In addition,stem cell-mediated exosomes and mitochondrial transfer demonstrate another novel mechanism

Stem cells exert their therapeutic effect by homing, differentiation and paracrine stimulation (Fig. 3). Stem cells that spontaneously migrate to the injured ovary are induced by multiple factors to adhere and proliferate. Current studies indicate that the therapeutic effect of stem cell transplantation may be mediated by paracrine mechanisms [64]. Paracrine signaling involves the secretion of multiple biologically active molecules, including growth factors, cytokines, regulatory factors, and signal peptides by surrounding cells to influence adjacent cells. This process improves the condition of damaged ovaries through anti-apoptosis, anti-fibrosis, angiogenesis, anti-inflammation, and immune regulation.

Further investigation of paracrine signals indicates that stem cells secrete exosomes to mediate their functions. Exosomes are extracellular vesicles that carry proteins, mRNA, and microRNA (30-130 nm in size) [65]. These vesicles mediate cell-to-cell communication via target cell internalization, ligand-receptor interaction, or lipid membrane fusion [66]. Depending on their source cells, exosomes can initiate repair and regeneration processes to restore critical cellular functions and maintain tissue homeostasis thereby.

Stem cell-mediated mitochondrial transfer is another novel mechanism developed in recent years. Mitochondria are transferred from stem cells to adjacent cells through tunnel nanotubes, formed by pro-inflammatory cytokines that drive skeletal rearrangement in stem cells. It has also been recently reported that inflammation-driven transfer of mitochondria from stem cells to oocytes may rescue oocyte quality and embryo development [67, 68].

#### Stem cell therapy for POI

Although ESCs show unlimited potential for differentiation, clinical use of ESCs is limited. This is because the use of ESCs raises challenging ethical issues, such as the destruction of blastocysts. By contrast, iPSCs, which are prepared by reprogramming human somatic cells, may be used without any ethical issues. Yamashiro et al. confirmed that human iPSCs could differentiate into human primitive germ-like cells (hPGCLCs) in vitro. More importantly, hPGCLCs cultured under defined conditions differentiated into oogonia/gonocyte-like cells [69].

Significant progress has been made in the treatment of POI with MSCs (Table 1). Previous studies have reported that bone marrow stem cells (BMSCs) injected into mice reduced FSH levels and increased estrogen levels and follicle numbers, by increasing the secretion of vascular endothelial growth factor (VEGF) [70]. Furthermore, BMSCs could inhibit GC apoptosis by downregulating cyclin-dependent kinase inhibitor 1A (P21) and Bcl-2-associated X protein (Bax), and upregulating the c-myc proto-oncogene mRNA [71].

**Table 1:** Summary of stem cell therapy for POI

Types	Species and POI model	Mechanisms	Method of administration	Outcome	Reference
iPSCs	ICR mice	differentiation function	cultured and induced in vitro	differentiate into oogonia/gonocyte-like cells	[69]
BMSCs	cyclophosphamide-induced ovarian failure in rabbits	increased the secretion of VEGF	injected intravenously in the ear veins	reduced FSH level increased serum estrogen level and follicle number	[70]
BMSCs	cisplatin-induced apoptosis of GCs/ aging rats	downregulated P21 and Bax, upregulated the c-myc proto-oncogene mRNA	co-culture in vitro/injected via vena caudalis	inhibited GCs apoptosis	[71]
ADSCs	cisplatin-induced ovarian failure in mice	induced angiogenesis	intraovarian injected	restored the number of ovarian follicles and corpus luteum	[72]
MenSCs	epirubicin-induced GCs injury/ cisplatin-induced ovarian failure in mice	protected GCs from apoptosis and increased secretion of fibroblast growth factor 2	co-culture in vitro/injected via vena caudalis	improved POI	[73, 74]
PMSCs	the pZP3-induced POI mice model	reduced follicular atresia and GCs apoptosis	injected via vena caudalis	increased serum AMH and estrogen levels	[75]
PMSCs	the pZP3-induced POI mice model	regulated Th17/Tc17 and Th17/Treg cell ratios by PI3K/Akt signal pathway	intraovarian injected	restored ovarian function	[76]
PMSCs	ovariectomized rat model	upregulated antioxidant factors	injected through the tail vein	restored ovarian function	[77]
PMSCs	the pZP3-induced POI mice model	inhibited endoplasmic reticulum stress inositol-demanding enzyme 1 signaling pathway	injected through the tail vein	reduced GCs apoptosis	[78]
UC-MSCs	cisplatin-induced ovarian failure in rat	transform growth factor-β/Smad3 signaling pathway	injected through the tail vein	regulated the differentiation of ovarian stromal cells	[79]
AFSCs	gilt	differentiatived potential	co-culture in vitro	differentiated into primordial follicle oocytes in vitro	[80]
AMSCs	cyclophosphamide-induced ovarian failure in rat	reduced the expression of inflammatory cytokines	injected through the tail vein	inhibited ovarian inflammation	[81]
exosomal miR-644-5p derived from BMSCs	cisplatin-induced ovarian failure in mice	targeted the regulation of p53	injected through the tail vein	suppressed GCs apoptosis	[82]
exosomal miR-144-5p derived from BMSCs	cyclophosphamide-induced ovarian failure in rat	target PTEN	injected intraperitoneally	inhibited GCs apoptosis	[83]
miR-21 derived from BMSCs	phosphamide mustard- induced apoptosis of GCs/ cyclophosphamide-induced ovarian failure in rat	downregulated PTEN and PDCD4	co-culture in vitro/ injected into the bilateral ovaries	repaired ovarian structure and function	[84]
ADSC-derived exosomes	cyclophosphamide-induced ovarian failure in mice	regulated the SMAD pathway	injected into the ovaries	oogenesis and the proliferation of granular cells	[85]
exosomal miRNA-17-5p derived from hUC-MSCs	cyclophosphamide-induced ovarian failure in mice	regulated SIRT7 signaling pathway	injected into the ovaries	improved ovarian function	[86]
exosomal miR-10a/ and miR-146a derived from AFSCs	cyclophosphamide-induced ovarian failure in mice	inhibited follicular atresia	transplanted into the ovaries	increased GCs survival	[87]
exosomal miR-320areleased from human AMSCs	cyclophosphamide-induced ovarian failure in mice	regulated Sirtuin4	injected into the ovaries	delay ROS generation in POI	[88]
collagen caffold+ ADSCs	tripterygium glycosides induced ovarian failure in rat	enhance the retention of ADSCs in target organs	injected into the ovaries	increase follicle counts	[89]
collagen caffold+ UC-MSCs	POI patients	phosphorylation of FOXO3a and FOXO1	intraovarian injected/	activate follicles in dormant ovaries	[90]
alginate+ ADSCs	mice	secrete cytokines	co-culture in vitro	support follicle genesis, survival, and maturation in vitro	[91]

Adipose-derived stem cell (ADSCs), which are pluripotent and easy to access, are ideal therapeutic cells. A study demonstrated that ADSC implantation induces angiogenesis, restores the number of follicles and the corpus luteum, and repairs ovarian damage thereby [72]. Menstrual blood-derived mesenchymal stem cells (MenSCs) are increasingly attracting attention, due to the controversy regarding the non-ethical nature of their usage, as also low immune rejection and toxicity [100]. Previous studies have showed that MenSCs protected GCs from apoptosis and increased the secretion of fibroblast growth factor 2, thereby improving POI [73, 74].

In addition, placenta-derived mesenchymal stem cells (PMSCs) transplantation significantly increased serum AMH and estrogen, while dramatically reducing follicular atresia and GC apoptosis [75]. Based on the results of recent studies, the therapeutic mechanisms underlying the alleviation of POI by PMSCs may include: (i) restoration of ovarian function via the regulation of Th17/Tc17 and Th17/Treg cell ratios by the PI3K/Akt signaling pathway; (ii) upregulation of antioxidant factors, which has been shown to restore ovarian function in ovariectomized rats; and (iii) reduction of GC apoptosis by inhibiting the endoplasmic reticulum stress inositol-demanding enzyme 1signaling pathway [76–78].

Umbilical cord mesenchymal stem cells (UC-MSCs) are widely used to treat POI. Human UC-MSCs inhibited ovarian fibrosis in POI rats by regulating the differentiation of ovarian stromal cells via the transforming growth factor-β/Smad3 signaling pathway [79]. Additionally, human amniotic fluid stem cells (AFSCs) have shown potential for differentiating into primordial follicle oocytes in vitro [80]. Amnion mesenchymal stem cells (AMSCs) also reduced the expression of inflammatory cytokines, which inhibited ovarian inflammation in rats with POI. Pretreatment of AMSCs using low-intensity pulsed ultrasound, augmented anti-inflammatory effects [81].

#### Cell-free therapy for POI

Treating POI with exosomes is associated with higher clinical safety, because immune rejection and the risk of vascular obstruction and tumor mutation can be avoided by using exosomes. A recent study revealed that exosomal miR-644-5p derived from BMSCs targeted the regulation of p53 to suppress the apoptosis of GCs, thus alleviating POI [82]. In 2020, Yang et al. reported that BMSCs-derived exosomal miR-144-5p relieved POI by targeting PTEN to inhibit GC apoptosis [83]. In addition, overexpression of miR-21, a key miRNA that regulates apoptosis in BMSCs, repaired ovarian structure and function in rats, by downregulating PTEN and the programmed cell death protein 4 (PDCD 4) [84].

ADSC-derived exosomes restored ovarian function in POI patients by regulating the SMAD pathway [85]. Besides, exosomal miRNA-17-5p derived from UC-MSCs improved ovarian function by regulating SIRT7 [86]. Moreover, exosomal miR-10a and miR-146a derived from AFSCs were essential for inhibiting follicular atresia and increasing GC survival, wherein miR-10a plays a leading role [87]. Ding et al. reported that exosomal miR-320a, released from human AMSCs, delayed ROS production in POI by regulating Sirtuin4 [88].

#### Biomaterial strategies for POI

Stem cell transplantation leads to extreme cell loss. Consequently, biomaterials including collagen, alginate, and hyaluronic acid (HA) have to be introduced. Collagen is essential for maintaining biological activity. He et al. created collagen-rich, biomimetic 3D shells via microfluidic encapsulation, where follicle culture with these biomimetic capsules helped these to develop into the antral stage [92]. Su et al. used a collagen scaffold to enhance the retention of ADSCs in target organs [89]. Similarly, Ding et al. reported that UC-MSCs on collagen scaffolds activated follicles via the phosphorylation of FOXO3a and FOXO1 [90].

Alginates may be used for drug delivery due to their biocompatibility, non-immunogenicity, and hydrophilicity. These have been used for the culture of secondary and pre-antral follicles [93]. ADSCs co-encapsulated with ovarian follicles in an alginate-based 3D culture system supported follicle genesis, survival, and maturation in vitro, via the secretion of cytokines [91]. HA is another widely used biological material. Certain tissues, such as those of the uterus and ovaries, that have HA receptors are amenable to targeted therapy [94]. Importantly, HA levels in POI patients are relatively low, and thus HA supplementation can be effectively used to prevent chemically induced ovarian injury and improve ovarian function [95].

#### Safety and optimization measures for stem cell therapy

The safety of stem cells must be evaluated so that they can be standardized prior to clinical application. Tumorigenicity, immunogenicity, and heterogeneity of ESCs and iPSCs typically limit their application. Among these, tumorigenicity presents the most concerning issue. The factors associated with tumorigenesis are as follows: (i) residual undifferentiated and/or immature cells present in stem cell populations lead to incorrect patterning; (ii) reprogramming factors specific to induced iPSCs promote tumorigenesis; and (iii) genetic mutations induced by the expansion of stem cells in vitro may cause tumorigenicity [96]. Therefore, effective methods that ensure directional differentiation, strict procedures of purification, and careful selection of cell lines are of great significance in regard to the safety of this therapy.

With respect to immunogenicity, autologous stem cell transplantation is an ideal choice to eliminate rejection. However, allogeneic transplantation is preferred to autologous transplantation due to time and cost constraints. Although immunosuppressive agents are currently used to overcome allograft rejection, their side effects, such as higher infection risk, cannot be disregarded. The human leukocyte antigen (HLA), an important component of the human immune system, is critical for immune rejection. Therefore, researchers are attempting to circumvent immune rejection by matching HLA haplotypes or inactivating HLA genes. Furthermore, as no two cells are similar, researchers are focused on overcoming heterogeneity by converting stem cells to their naive and ground states. However, further research is needed to address these challenges.

Clinical trials conducted worldwide have indicated that MSCs with low immunogenicity and tumorigenicity are safe sources of stem cells [97]. However, the therapeutic effects of these cells are still affected by multiple factors, such as the quality and quantity of cell products amplified in vitro, route of injection, optimal dose, and timing of the treatment. To date, researchers and clinicians have not been able to address these issues effectively.

### PRP intra-ovarian infusion

#### Mechanisms of PRP in POI

Intra-ovarian infusion of PRP is another novel approach to the treatment of POI. PRP is composed of high concentrations of platelets obtained from the peripheral blood of patients via centrifugation [98]. The efficiency of PRP depends mainly on their α-granule content, which is highly enriched in proteins, hormones, and growth factors [99]. The release of bioactive proteins promotes cell proliferation and differentiation [101]. In addition, activated platelets release high concentrations of hormones and growth factors, which stimulate angiogenesis as well as anabolism and inflammation control, thereby rapidly promoting the healing and regeneration of tissues [102]. Importantly, GDF-9, implicated in the maturation potential of oocytes, and mutated in POI, is also present in PRP [103].

The mechanisms underlying the role of PRP in the treatment of POI remain elusive. Several studies have reported that PRP promotes the development of primitive and primary follicles into the presinus stage [104]. Thus, some studies have used AMH as a principal marker to evaluate the efficacy of PRP because AMH levels, which are mainly associated with preantral and antral follicles, appear to be relatively stable across the menstrual cycle [105, 106]. Furthermore, PRP can restore the ovarian microenvironment. More specifically, PRP prevented OS and reduce the oxidation state of the ovary in rat with ovarian ischemia-reperfusion injury [107]. PRP also successfully reduced degeneration and atresia in normal follicles, caused by ovotoxic chemicals, and accelerated angiogenesis [108].

#### Application of PRP therapy in POI

Clinical application of PRP in human ovaries was first introduced by Pantos et al. [109]. In this study, eight peri-menopausal women were treated with intra-ovarian PRP injections. Their results showed that the menstrual cycle and oocyte retrieval in all patients had recovered following in vitro fertilization (IVF) treatment. However, a small proportion of patients and their previous ovarian reserves were not recorded, limiting the interpretation of these results. In addition, PRP reportedly exerts positive effects on ovarian vascularization. For example, PRP was used during autologous ovarian transplantations to increase the vascular density of grafts [110].

Sfakianoudis et al. (2018) reported that a patient with POI receiving PRP treatment became pregnant following a natural IVF cycle [111]. Sfakianoudis et al. (2020) further reported pilot data pertaining to PRP treatment in POI, which indicated poor ovarian response (POR), peri-menopausal, and menopausal women [99]. The results of this study indicated that the levels of both AMH and AFC were increased while those of FSH and luteinizing hormone were decreased in all patients tested. These research results indicated that PRP therapy effectively restored ovarian functionality and hormonal profile. In another study, researchers evaluated the effect of this therapy on ovarian reserves and IVF outcomes in 311 patients with POI. These results indicated that 23 patients (7.4%) conceived spontaneously after treatment with PRP [102]. In order to improve clinical effect, Chao-Chin et al. injected PRP combined with gonadotropin into the ovarian stroma. Following this treatment, the patient, who had responded poorly to gonadotropins earlier, resumed menstrual periods and achieved successful conception [112].

#### Risk and countermeasures of PRP therapy

Advantages of autologous PRP intra-ovarian infusion include easy handling, good storage properties, and low immunogenicity. However, the potential risk associated with PRP therapy mainly include infection, intense cell proliferation events, and unknown detrimental effects on the embryo. In terms of infection risk, a few PRP samples were found to be positive for microbial growth [113]. In addition, other adverse events such as infections have been linked to PRP [114]. Therefore, detection and inactivation of blood borne pathogens in samples is vital, despite the fact that some PRP preparations have been found to exhibit antimicrobial properties.

Growth factors derived from PRP govern cell proliferation and differentiation [115]. However, intense cell proliferation events may induce malignancy due to differentiation of stem cells within the ovaries [116]. The final risk associated with this therapy involves detrimental effects exerted on the embryo by high concentrations of hematopoietic cells introduced into the implantation environment by PRP [117]. Thus, further studies are required to provide solid evidence that confirms the safety of PRP therapy.

### MicroRNAs: the future direction of POI treatment

MicroRNAs (miRNAs) are short, 18–24 nucleotides long, non-coding RNAs [118]. These regulate cell proliferation, differentiation, and apoptosis in normal and pathological processes [119]. The expression levels of miRNAs in reproductive tissues appears to be linked to fertility potentials and embryo developmental capacities [120]. Furthermore, miRNAs play a regulatory function in folliculogenesis and oocyte maturation [119] and are detected in plasma, serum, and urine. Currently, plasma miRNAs are considered as promising potential biomarkers for a series of cancers and other diseases.

A miRNA microarray analysis conducted by a previous study indicated that 22 miRNAs were significantly upregulated, while 29 were significantly downregulated in POI patients [121]. Several critical miRNAs in POI have been described. MiR-23a and miR-27a are significantly upregulated in the plasma of POI patients [121, 122]. Reportedly, miR-23a induces GC apoptosis by downregulating the X-linked inhibitor of apoptosis proteins and increasing caspase-3 cleavage [122]. These two miRNAs induce GC apoptosis by targeting SMAD5, which activatse the Fas ligand-Fas pathway in vitro [123]. Moreover, a miR-27a mimic sequence transfected into GCs reduced oocyte maturation in mouse follicles [124].

MiR-127-5p is significantly upregulated in the plasma of patients with biochemical POI (bPOI). Biochemical POI refers to elevated FSH levels accompanied by normal menstrual cycle. A study has shown that miR-127-5p inhibits the proliferation of GCs and impairs the ability of DNA damage repair by targeting the POI high mobility group box2 gene [125]. Intriguingly, miR-127-5p was also upregulated in the plasma of bPOI patients, and thus, miR-127-5p has been proposed as a biomarker for bPOI.

Intensive research studies have laid the foundation for using miRNAs in POI therapy. A study found that miRNA-146b-5p overexpression attenuates POI in mice by specifically downregulating p38-Mapk14 and inhibiting γH2A.X phosphorylation. The novelty of this study is that it researched poly (lactic-co-glycolic acid) (PLGA) nanoparticles as a carrier to achieve successful delivery of miRNA-146b-5p. Next, these researchers cultured GCs, derived from mouse POI model, in a medium supplemented with PLGA cross-linked miRNA-146b-5p. The GCs displayed downregulated p38-Mapk14 and alleviated aging.

## Conclusion

POI patients have unique needs that require special attention. Different therapeutic strategies that focus on rescuing ovarian function open new opportunities for women with POI. Knowledge regarding various aspects of related fields will enhance the possibilities of treating this disease. Several issues need to be resolved prior to optimization of POI management. Specifically, HRT still remains the central element in the treatment of POI. Clinicians bear responsibility for informing patients that untreated POI may increase the risk for premature death and cardiovascular disease, and should consider the patient’s condition, needs, and preferences during the decision-making process to adjust the formulation, dosage, and duration of use according to individual patient needs. Moreover, an evaluation of the clinical effect exerted by the assigned HRT on the patient needs to be performed.

Although current studies support the development of IVA, this technique is still in its formative stage. Residual follicles form the premise of IVA technology. Therefore, it is necessary to develop novel methods, including imaging techniques such as optical coherence tomography, that help localize residual follicles. In addition, mechanisms underlying fragmentation that disrupts the Hippo pathway, targeting of additional pathways, or other factors that accelerate PF activation, warrant further investigation. Finally, the culturing system of activated follicles should be optimized by incorporating compatible hormonal, paracrine environments and mechanical characteristics.

Currently, due to the scarcity of pertinent studies, strong evidence that demonstrates the effect and safety of mitochondrial activation techniques cannot be produced. Mitochondrial nutrient and transfer therapy continue to be explored. A better understanding of the mechanisms underlying the role played by mitochondrial dysfunction in ovarian aging should be obtained via further research. Revealing the determinants of mitochondrial dysfunction may enhance the development of targeted intervention.

Stem cell therapy has shown promising results against POI. Utilization of exosome replacement cell itself is sufficient to overcome tumorigenesis and immunogenicity of stem cell. Importantly, various biomaterials can be used to deliver stem cell or exosomes, in order to increase the retention and survival rates of stem cells or exosomes in target organs. The future direction of research appears to tilt towards the application of exosomes combined with natural biomaterials. However, in this respect, the biosafety, biodegradation, and biocompatibility of new materials cannot be disregarded.

PRP may be considered as a putative alternative strategy for treating POI. Exhaustive investigation of the safety of this therapy prior to clinical practice is critical. Clinicians offering PRP should confirm platelet and growth factor concentrations, as well as constitution and detection of blood borne pathogens in samples. Moreover, further studies that include appropriate control groups, which help evaluate the efficacy of this technique, should be initiated. The next logical step would be the careful implementation of precisely designed, large scale, randomized clinical trials.

In this review, we propose miRNAs as a potential treatment option for POI. An in-depth understanding of the role of miRNAs in POI would be advantageous in the treatment of ovarian diseases. It is possible to target POI via therapies involving transfected mimics or inhibitors of specific miRNAs. In addition, the prediction and validation of upstream regulators and downstream target genes of miRNAs may also contribute towards the understanding and treatment of POI.

These new therapies will allow clinicians to perform high-quality interventions in the treatment of POI. Notably, a majority of current research on these therapies has been performed on animal models. Considering the vast differences between humans and animals, precise experiments, designed for vertebrates at evolutionary stages varying from lower to higher levels, may ensure that these new technologies can be safely and effectively applied to humans. Furthermore, the sense of balance between patience and caution should be fine-tuned when applying various therapies against POI.

## Data Availability

Not applicable.

## Abbreviations

POI: Primary ovarian insufficiency
POF: Premature ovarian failure
FSH: Follicle-stimulating hormone
AMH: Anti-Mullerian hormone
AFC: Antral follicle count
HRT: Hormone replacement therapy
IVA: In vitro activation
PRP: Platelet-rich plasma
TD: Transdermal patches
HA: Hyaluronic acid
RBCs: Red blood cells
PFs: Primordial follicles
PTEN: Phosphatase and tensin homolog
PI3K: Phosphatidylinositol-3-kinase
Akt: Protein kinase B
FOXO3: Forkhead box O3
PIP2: Phosphatidylinositol-4,5-bisphosphate
PIP3: Phosphatidylinositol-3,4,5-triphosphate
PDK1: Phosphatidylinositol-dependent kinase 1
TSC2: Tuberous sclerosis 2
mTORC1: mammalian target of rapamycin complex
S6K: S6 kinase
rpS6: ribosomal protein S6
4E-BP1: 4E binding protein 1
YAP: Yes-associated protein
TAZ: Transcriptional co-activator PDZ-binding motif
BIRC: Baculoviral IAP repeat-containing
BMPs: Bone morphogenetic proteins
GDF-9: Growth and differentiation factor 9
GCs: Granulose cells
ECM: Extracellular matrix
mtDNA: mitochondrial DNA
ATP: Adenosine triphosphate
ROS: Reactive oxygen species
POLG: Polymerase gamma
OS: Oxidative stress
MFN2: Mitofusin 2
Drp1: Dynamin-related protein 1
CoQ10: Coenzyme Q10
GV: Germinal vesicle
PNT: Pronuclear transplantation
AUGMENT: Autonomous germline mitochondrial energy transfer
ESCs: Embryonic stem cells
ASCs: Adult stem cells
iPSCs: Induced pluripotent stem cells
MSCs: Mesenchymal stem cells
hPGCLCs: Human primitive germ-like cells
BMSCs: Bone marrow stem cells
VEGF: Vascular endothelial growth factor
P21: Cyclin-dependent kinase inhibitor 1A
Bax: Bcl-2-associated X protein
ADSCs: Adipose-derived stem cell
MenSCs: Menstrual blood-derived mesenchymal stem cells
PMSCs: Placenta-derived mesenchymal stem cells
AMSCs: Amnion mesenchymal stem cells
UC-MSCs: Umbilical cord mesenchymal stem cells
AFSCs: Amniotic fluid stem cells
PDCD 4: Programmed cell death protein 4
HLA: Human leukocyte antigen
IVF: In vitro fertilization
POR: Poor ovarian response
miRNAs: micrornas
bPOI: biochemical POI
