# Correction: Therapeutic options for premature ovarian insufficiency: an updated review

**DOI:** 10.1186/s12958-023-01133-2

**Published:** 2023-09-04

**Authors:** Qiao-yi Huang, Shao-rong Chen, Jia-ming Chen, Qi-yang Shi, Shu Lin

**Affiliations:** 1 Department of Gynaecology and Obstetrics, The Second Affiliated Hospital of Fujian Medical University, No.34 North Zhongshan Road, Quanzhou, 362000 Fujian Province China; 2 Centre of Neurological and Metabolic Research, The Second Affiliated Hospital of Fujian Medical University, No.34 North Zhongshan Road, Quanzhou, 362000 Fujian Province China; 3 Diabetes and Metabolism Division, Garvan Institute of Medical Research, Darlinghurst, 384 Victoria Street, Sydney, Australia NSW 2010

**Correction: Reprod Biol Endocrinol 20, 28 (2022)**.

10.1186/s12958-022-00892-8.

Following publication of the original article [1], the authors reported an error in the reference list. The author names found in reference 116 is not correct. The correct details of the reference is provided below.

Sills ES, Wood SH. Autologous activated platelet-rich plasma injection into adult human ovary tissue: Molecular mechanism, analysis, and discussion of reproductive response. Biosci Rep. 2019;39(6):BSR20190805. 10.1042/BSR20190805.

The original article [1] has been updated.
